# Effects and Responsiveness of a Multicomponent Intervention on Body Composition, Physical Fitness, and Leptin in Overweight/Obese Adolescents

**DOI:** 10.3390/ijerph18147267

**Published:** 2021-07-07

**Authors:** Leticia Borfe, Caroline Brand, Letícia de Borba Schneiders, Jorge Mota, Claudia Regina Cavaglieri, Neiva Leite, Jane Dagmar Pollo Renner, Cézane Priscila Reuter, Anelise Reis Gaya

**Affiliations:** 1 Graduate Program in Human Movement Sciences, Federal University of Rio Grande do Sul, Porto Alegre 90690-200, Brazil; borfe.leticia@gmail.com; 2 Graduate Program on Health Promotion, University of Santa Cruz do Sul, Santa Cruz do Sul 96816-501, Brazil; carolbrand@hotmail.com (C.B.); leticiaschneiders12@gmail.com (L.d.B.S.); 3 Research Center in Physical Activity, Health and Leisure, Faculty of Sports, University of Porto, 4200-450 Porto, Portugal; jmota@fade.up.pt; 4 Department of Adapted Physical Activity Studies, University State Campinas, Campinas 13083-851, Brazil; cavaglieri@fef.unicamp.br; 5 Department of Physical Education, University of Paraná, Curitiba 81690-100, Brazil; neivaleite@gmail.com; 6 Department of Life Sciences and Graduate Program on Health Promotion, University of Santa Cruz do Sul, Santa Cruz do Sul 96816-501, Brazil; janerenner@unisc.br; 7 Department of Health Sciences and Graduate Program on Health Promotion, University of Santa Cruz do Sul, Santa Cruz do Sul 96816-501, Brazil; cezanereuter@unisc.br; 8 Graduate Program in Human Movement Sciences, School of Physical Education, Federal University of Rio Grande do Sul, Porto Alegre 90690-200, Brazil

**Keywords:** obesity, physical exercise, youth, cardiorespiratory fitness, inter-individual variability

## Abstract

Physical exercise reduces the biochemical markers of obesity, but the effects of multicomponent interventions on these markers should be explored. The present study aimed to elucidate how overweight/obese adolescents respond to a multicomponent program approach on body composition, physical fitness, and inflammatory markers, using a quasi-experimental study with 33 overweight/obesity adolescents (control group (CG) = 16; intervention group (IG) = 17). The intervention consisted of 24 weeks with physical exercises and nutritional and psychological guidance. Both groups were evaluated at the pre/post-intervention moments on body mass index (BMI); body fat (%Fat); waist circumference (WC); waist/hip ratio (WHR); waist-to-height ratio (WHtR), cardiorespiratory fitness (CRF); abdominal strength, flexibility; leptin; interleukin 6; interleukin 10; and tumor necrosis factor-alpha. Mixed-analysis of variance and generalized estimation equations were used for statistical analysis. There was an interaction effect between groups and time on %Fat (*p* = 0.002), WC (*p* = 0.023), WHR (*p* < 0.001), WHtR (*p* = 0.035), CRF (*p* = 0.050), and leptin (*p* = 0.026). Adolescents were classified as 82.4% responders for %Fat, 70.6% for WC, 88.2% for WHR, and 70.6% for CRF. Further, there was an association between changes in %Fat (*p* = 0.033), WC (*p* = 0.032), and WHR (*p* = 0.033) between responders and non-responders with CRF in the IG. There was a positive effect on body composition, physical fitness, and leptin. In addition, reductions in body composition parameters were explained by CRF improvements.

## 1. Introduction

Child and youth obesity currently has alarming proportions worldwide [1]. Physical inactivity and sedentary behaviors have increased among all ages groups [2] and have been identified as a risk factor for the early development of overweight and consequently metabolic disorders [3]. The increase in body fat contributes to the development of chronic sub clinical inflammation, resulting in a high release of pro-inflammatory cytokine as tumor necrosis factor-alpha (TNF-α), interleukin-6 (IL-6), leptin and C-reactive protein (CRP) and reduction of anti-inflammatory cytokine, such interleukin-10 (IL-10) and adiponectin in both youth and adults [4,5]. Called a low-grade inflammatory state, this increase in the systemic inflammatory level has been linked to the progression of cardiometabolic disease risks, such as insulin resistance, visceral obesity, metabolic syndrome and type 2 diabetes, with a higher incidence of pro-inflammatory cytokines secreted by macrophages with M1 phenotype [6,7].

Adolescence is a period of physical, cognitive, and social development, and at this time, the behaviors developed can last into adulthood. It is in this phase of increasing autonomy that health-related behaviors may be more susceptible to change. Therefore, preventive and rehabilitation interventions applied in this “window of opportunity” can be more effective in promoting changes to healthy behavior and, thus, in improving health throughout life [8]. In this regard, evidence suggests that behavioral-based interventions with a multidisciplinary approach, which includes individual and social aspects, are effective in improving the health status of overweight young people [9,10].

The current literature highlights that programs that include regular physical training, in the treatment of excess weight and its comorbidities, demonstrate improvements in physical fitness and body fat reduction, which may induce physiological, endocrine, and cardiovascular adaptations [11,12]. In addition, physical training promotes an anti-inflammatory state, which in the long term prevents the development of chronic diseases [13] and contributes to the increase in cardiorespiratory fitness (CRF), which, therefore, can contribute to the immunoregulatory role, helping to maintain low levels of inflammatory mediators [14]. In this sense, the program developed in this study is relevant in terms of encouraging healthy lifestyle habits such as regular physical exercise or sports to remain active throughout life.

However, most of the results based on obesity intervention programs are highlighted by comparing the groups using mean values. Few studies report an individual prevalence of responses, especially in children and adolescents. Additionally, although some studies have reported such prevalence of responders, they highlight results on cardiometabolic risk factors including adiposity, glycemic and lipid profile [8,10], levels of fat and liver enzymes [15] and components of metabolic syndrome in adults [16]. Nonetheless, the impact on circulating inflammation markers of obesity in adolescence is not yet fully understood.

Likewise, it is not yet clear what are the characteristics of an intervention program that must be taken into consideration for the treatment of obesity in adolescents, including the type of exercise, duration, and intensity, to exert a beneficial effect on the individual responsiveness. Therefore, this study aims to elucidate how overweight and obese adolescents respond to a multicomponent program, including different types of physical exercises (resistance, aerobic, aquatic, and sports exercises), nutrition and psychological approach on body composition, physical fitness, and circulating inflammatory markers, as well as to determine the individual prevalence of responsiveness on these parameters and the main mediators associated with the positive effects.

## 2. Materials and Methods

### 2.1. Study Design

This quasi-experimental study was part of a larger study called: “Obesity in elementary school students: an interdisciplinary intervention study-Phase III”, developed with overweight and obese adolescents of both sexes, aged between 10 and 17 years. The research was approved by the Research Ethics Committee of the University of Santa Cruz do Sul (UNISC), under the number 1,498,338. In addition, it is registered as a clinical trial under Protocol ID: 54985316.0.0000.5343. All the participants were informed about the research procedures, parents or legal guardians of the adolescents signed free and informed consent forms, and the adolescent signed the assent form.

### 2.2. Participants

The initial sample of this study was obtained from the cross-sectional research “Health of students-Phase III”, carried out at the University of Santa Cruz do Sul, in which overweight and obese adolescents were identified through body mass index (BMI) assessments, classified according to the World Health Organization percentile curves [17]. Of these, 172 adolescents, all residents close to the university, were invited to participate in the research on a voluntary basis. The project was publicized by the media (newspaper, radio, and internet) in order to increase the number of participants.

As inclusion criteria, adolescents needed to present the signed free and informed consent form and informed consent form for the underage child; be aged between 10 to 17 years; perform blood collection; not have participated in another physical exercise program simultaneously with the intervention project and in the last six months and be able to participate only in regular physical education classes. The exclusion criteria were a frequency of participation in the intervention program below 60%, or presenting incomplete data in physical tests or blood tests. The sample size calculation was performed in G*Power 3.1.9.7 [18] Anova: repeated measures, within-between interaction, was considered a test power of 0.95, an effect of 0.30, and a significance level of 95%, requiring at least 13 subjects for the control group (CG) and 13 subjects for the intervention group (IG).

**Figure 1:**
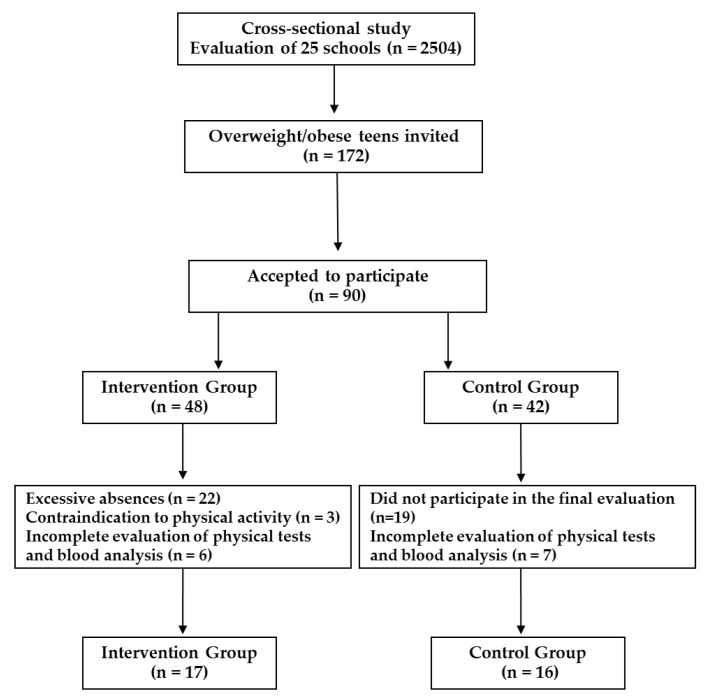
Sample’s flow diagram.

Initially, 90 adolescents agreed to participate and were allocated by convenience criteria into two groups: Intervention Group (IG, *n* = 48) and Control Group (CG, *n* = 42). The multicomponent intervention program had an initial participation of 48 members in the IG. However, over the 24 weeks of intervention, there was a sample loss of 25 adolescents, 22 of them due to excessive absences; 3 because they presented some type of contraindication to performing physical activity during the program, and another 6 adolescents were excluded because they did not present complete data on blood tests or physical tests. In the CG, 42 adolescents participated in the initial evaluation, but at the end of the intervention program, only 23 performed the post-test evaluation. Afterward, seven adolescents were excluded for not presenting complete data on physical tests or blood tests. The sample flow diagram can be seen in Figure 1.

### 2.3. Multicomponent Intervention Program

The multicomponent intervention program consisted of sessions of physical exercises, activities of nutritional guidance, and psychological counseling. This program was developed from April to September, on alternate days, Mondays, Wednesdays, and Fridays lasting 2 h and totaling 70 meetings in 24 weeks. The activities were developed in the afternoon after the school hours of the participants. The sessions were held by Physical Education professionals, a nutritionist, and a psychologist along with scientific initiation fellows from the Physical Education, Nutrition, and Psychology courses from UNISC. All program activities were carried out at the facilities of UNISC.

### 2.4. Physical Exercise Intervention

Physical exercise sessions took place 3 times a week for 24 weeks, being taught by the same Physical Education teachers. Each session included three phases: warm-up, training (aerobic, resistance, pre-sport, or water recreation) and relaxation/stretching activities. Monday’s sessions consisted of warming up, stretching, pedagogical processes and sports activities, and the classes were programmed with sport variation (football, volleyball, handball, basketball, futsal, dance, jiu-jitsu). On Wednesday, the session covered activities with a functional and resistance exercise circuit, respiratory re-education, and postural correction exercises. On Fridays, varied water activities were offered in a heated pool, which included swimming initiation, aqua aerobics, and recreational games.

The intensity of the activity developed was controlled by heart rate (HR) monitors (Polar Cardiac Monitor-FT1^®^, Finland) and the training intensity was defined to induce HR between 50% and 70% of maximum HR, considering a Karvonen equation (HR maximum = 220-age). Mandatory requirements and requirements were gradually increased.

### 2.5. Nutritional Education Intervention

The nutritional orientation sessions were developed by a nutritionist and trainees of the nutrition course, taking place once a week, with an average duration of 1 h. These sessions had a central focus on food re-education with educational activities on foods, adequate portions, products, health risks, ways of replacing them, or reducing the consumption of fats, sugars, and sodium in the daily diet. Several methodologies were addressed, such as lectures, conversation circles, delivery of educational materials, interactive games, and practical classes with healthy recipes at the University’s Dietetic Technique laboratory. Other information about the nutritional guidelines applied in this intervention can be found in the study of Poll et al. [19].

### 2.6. Psychological Intervention

The psychological intervention took place through guidance and group cognitive training, carried out by a psychologist and interns from the psychology course. The sessions took place once a week with an average duration of 50 min. Cognitive techniques were used to recognize and restructure dysfunctional thoughts and relaxation techniques. The program was carried out through group sessions with updates, psychoeducation about the techniques, execution of the technique worked on the day, and prescription of the homework. More information about psychological intervention can be read in the study by Lüdtke et al. [20].

### 2.7. Anthropometric Measures and Body Composition

The height of the adolescents was measured using a stadiometer coupled to a properly calibrated scale (Filizola^®^), which was used to verify body weight. BMI was determined by the formula BMI = weight/height² (kg/m²) and the results were subsequently classified by the WHO percentile curves [17], including overweight with percentile ≥ 85 and percentile < 97 and obesity percentile ≥ 97. To measure the waist circumference (WC) and the hip circumference (HC), a 1 mm inelastic measuring tape was used. Afterward, the waist-to-hip ratio (WHR) was obtained by dividing WC (cm) by hip circumference (cm). In addition, using the measurements already adjusted for WC and height, waist-to-height ratio (WHtR) was obtained by the ratio between WC and height to calculate body fat, tricipital, subscapular skinfolds were evaluated using a skinfold calibrator (Lange, Beta Technology Inc., Houston, TX, USA), following the equation proposed by Slaughter et al. [21].

### 2.8. Maturational Stages

Tanner’s adapted staging method [22] was applied as an indicator of sexual maturation. The self-assessment test was performed individually using images that represent the stage of development of pubic hair. In both sexes, pubic hair is evaluated for its characteristics, quantity, and distribution. Afterward, the stages of development are classified increasingly, from 1 to 5, with stage 1 being the infantile stage (impoverished) and stage 5 being the adult stage (post-pubertal). Thus, stages 2, 3, and 4 characterize the pubertal period. The stages were grouped into three categories: non-matured (initial development), continuous maturation (pubertal period), and matured (post-puberty).

### 2.9. Physical Fitness

To evaluate physical fitness, the protocols by the *Projeto Esporte Brasil* were followed [23]. To assess cardiorespiratory fitness, the 6-min running/walking test was performed on an official track with demarcated footage. Participants were previously recommended to wear light clothing and appropriate shoes, and at the time of the test, perform the largest number of laps running or walking on the track. The result was obtained in meters by the distance covered. Abdominal strength was verified through the abdominal resistance test, in which the subject is positioned in the supine position, knees flexed, and arms crossed over the chest performs the trunk flexion movement until touching the thighs with the elbows, returning to the initial position, the number of complete sit-ups performed in 1 min was noted. To determine the flexibility of the trunk, the sit and reach test with Wells bench was used, in which the subject obtained barefoot and with the heels touching the Wells bench, attempting to reach the ruler as far as possible. The results were measured in inches. This test was also recommended by Projeto Esporte Brazil [24].

### 2.10. Biochemical Assays

For blood analysis, 10 mL of blood was collected from the brachial vein after a 12-h overnight fast and 72 h after the last training session or evaluation. 5 mL was used in the vacant tube without anticoagulant (for analysis of biochemical, inflammatory, and metabolic indicators) and 5 mL of whole blood for the EDTA vacutainer tube (for blood count). All samples were collected, processed, divided into serum or plasma aliquots for analysis or stored at −80 °C. The blood was collected by a trained professional from the Exercise Biochemistry Laboratory, at UNISC. To determine leptin, interleukin-6 (IL-6), interleukin-10 (IL-10) and tumor necrosis factor-alpha (TNF-α) were studied in a serum sample (Luminex^®^ Platform, Life Technologies, Inc., São Paulo, Brazil) by the outsourced laboratory.

### 2.11. Statistical Analysis

To test the normality of the data, the Shapiro-Wilk test was used, and the homogeneity of variance was verified by the Levene test. The descriptive analysis of the dependent variables was performed by means and standard deviations (SD). The categorical variables were presented with absolute and percentage frequency to characterize the sample, and the difference between groups was determined through chi-square test. To compare the baseline variables between IG and GC, the one-way ANOVA test was used. The effect of the intervention was analyzed using generalized estimation equations (GEE), adjusted for maturation and sex, testing the main effects of group and times, as well as the respective interaction effects. Additionally, analyses were performed to derive mean relevant differences between groups. The effect size between pre and post-intervention was also calculated using the means and standard deviation of the groups through the Cohen’s *d*, with results classified as >0.2—small effect; >0.5—moderate effect; >0.8—large effect; >1.5—very large effect [25].

The prevalence of the responders was defined according to the magnitude of the size of the individual effect of each group (intervention and control), calculated by the calculation: Δ/SDpooled, where changes (Δ) were calculated as Post-intervention minus Pre-intervention values dividing by SDpooled. To define SDpooled, an equation was used: √((SD_1_^2^ + SD_2_^2^)/2), where SD_1_ is the base value and SD_2_ is the post-test value. The cutoff point was considered to be the one that presents an effect size (≥0.20 or ≤−0.20) after the intervention period. The difference in prevalence between groups was determined through logistic regression. The differences between CRF responders and non-responders for the results considered showed a difference in responsiveness between the CG and IG (i.e., body fat, WC, and WHR), which were examined by univariate linear models (ANCOVA) adjusted for baseline. All analyzes were performed using the Statistical Package for the Social Sciences, version 23.0 (SPSS, IBM Corp, Armonk, NY, USA) and the level of significance was set at α = 0.05 for all analysis.

## 3. Results

Table 1 shows the descriptive characteristics in both groups at baseline. The sample was composed of 48.5% boys and 51.5% girls. The IG presented higher mean values of WC (*p* = 0.04), WHR (*p* = 0.01) and IL-6 (*p* = 0.04), compared to CG.

Table 2 indicates the effect of time, group, and interaction on the indicators evaluated. It was found a significant interaction (time x group) on body fat (*p* = 0.001; *d* = −1.03), WC (*p* = 0.014; *d* = −0.81), WHR (*p* < 0.001; *d* = −1.41), WHtR (*p* = 0.022; *d* = −0.93), CRF (*p* = 0.034; *d* = 0.71) and leptin (*p* = 0.016; *d* = −0.85), in which IG presented an improvement on these parameters from the pre- to post-measures, compared to the CG.

**Figure 2:**
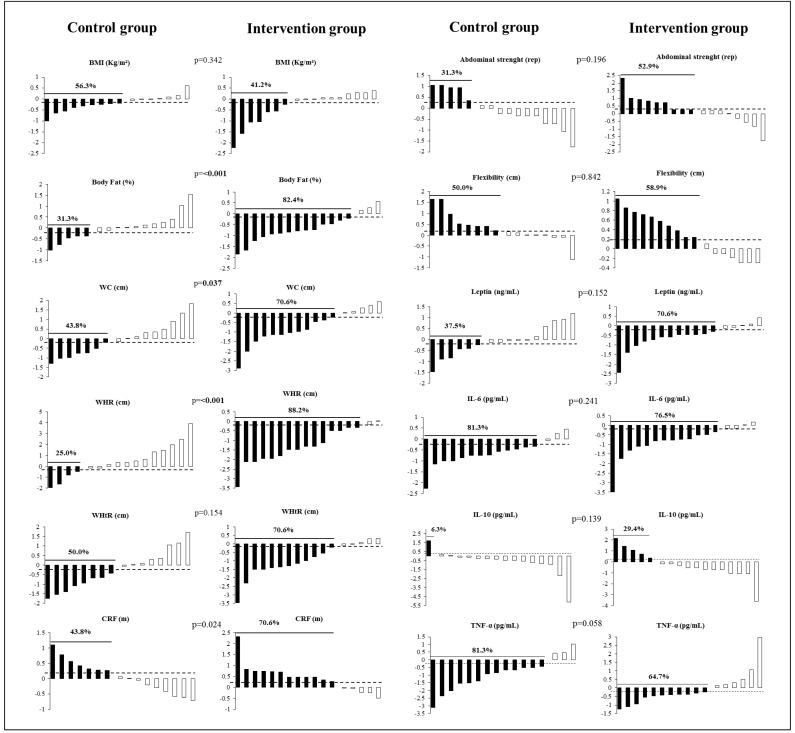
Prevalence of responders (*d*-Cohen > 0.2) on body composition, physical fitness, and inflammatory markers in the control group and intervention group after multicomponent intervention. ---- (respondent indicator line); Body mass index (BMI); body fat (%); waist circumference (WC); waist/hip ratio (WHR) and waist-to-hip ratio (WHtR); cardiorespiratory fitness (CRF); interleukin-6 (IL-6); interleukin-10 (IL-10); tumor necrosis factor-alpha (TNF-α). CG (*n* = 16) and IG (*n* = 17). The numbers represent the percentage of responders for each variable. *p* values indicate differences (χ^2^) in the prevalence of responders between control and intervention groups.

Figure 2 shows the individual prevalence and the distribution of responders and non-responders on body composition, physical fitness, and inflammatory markers after the multicomponent intervention. The data showed that the same adolescent could be classified as a responder for some variables but as non-responders for others, showing differences on individual variability towards intervention. As expected, it was found a significant difference in the prevalence of responders between CG and IG in body fat (CG = 31.3%; IG = 82.4%; PR (prevalence ratio) = 1.410; *p* < 0.001), WC (CG = 43.8%; IG = 70.6%; PR = 1.265; *p* = 0.037), WHR (CG = 25.0%; IG = 88.2%; PR = 1.586; *p* < 0.001) and CRF (CG = 43.8%; IG = 70.6%; PR = 1.284; *p* = 0.024). The other variables did not show differences in the prevalence of responders.

**Figure 3:**
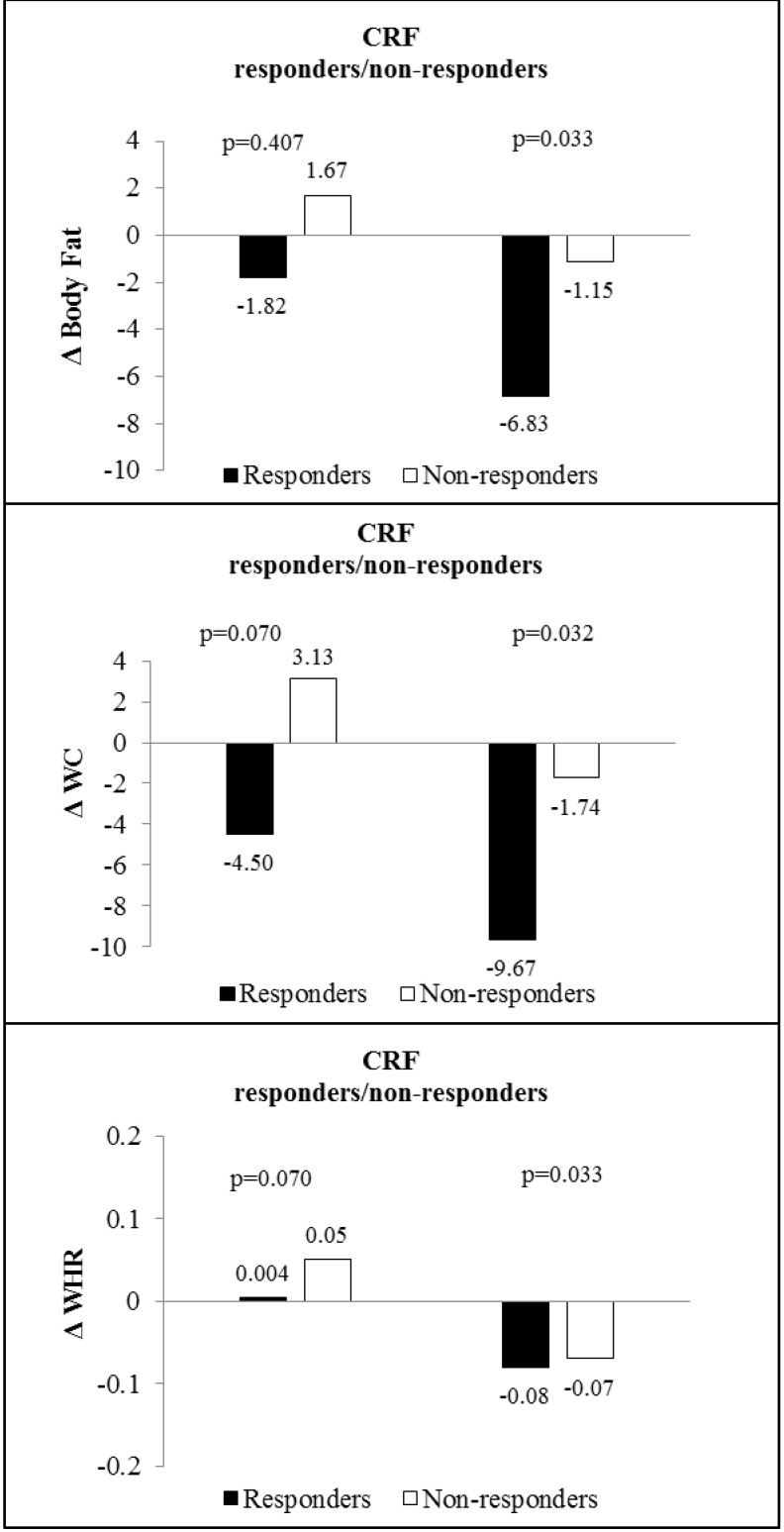
Differences in changes of body fat, waist circumference and waist to hip ratio between intervention and control groups in the responders and non-responders for cardiorespiratory fitness. Cardiorespiratory fitness (CRF); body fat (%); waist circumference (WC) and waist/hip ratio (WHR).

For the variables that showed a difference in responsiveness between CG and IG (body fat, WC, and WHR) we intend to understand the influence of CRF. Thus, differences in changes in body fat, WC, and WHR between responders and non-responders in CRF are presented in Figure 3. In the IG there were differences in the change of body fat, WC, and WHR between responders and non-responders for CRF, indicating that the adolescents classified as responders presented a reduction of 6.83% in body fat, 9.67 cm in WC, and 0.08 in WHR compared to the non-responders.

## 4. Discussion

The main findings of the present study indicate that: (1) there was an interaction effect between groups in body fat, WC, WHR, WHtR, CRF and leptin, in which IG presented an improvement on these parameters from the pre- to post-measures, compared to the CG; (2) The individual prevalence of responders showed that 82.4% of the adolescents were classified as responders on body fat, 70.6% for WC, 88.2% for WHR, and 70.6% for CRF in the IG. For those variables there was a significant difference in the prevalence of responders between IG and CG; (3) There was an association between the change in body fat, WC, and WHR between responders and non-responders for CRF in the IG.

Our data are aligned with previous studies namely on body composition, reporting decreased body fat, BMI, WC, WHtR, and WHR after physical exercise interventions, with durations ranging from three to six months and different intensities (moderate to vigorous) [10,15,26,27]. The present study also presented a significant improvement in the CRF levels of the IG after the intervention program. Similar results are reported regarding the improvement of aerobic fitness in a 12 to 14-week intervention with overweight and obese female adolescents [11,16], and in a study with 12 weeks of training interspersed with sports, swimming, and circuit training [28]. Moreover, the IG showed a significant reduction in leptin levels after the intervention program, which agrees with Zguira et al. [29] who conducted an 8-week individualized training program with obese adolescents. However, Lau et al. [30] did not show weight loss and decreased leptin levels in obese adolescents after physical training. Such divergent findings may be due to different exercise patterns and durations, the participants’ nutritional status, physical activity, leptin circadian rhythm, or the presence of genetic polymorphisms [31].

Although we have found important results in the aforementioned variables, there were no significant changes in the levels of IL-6, IL-10, and TNF-α between IG and CG after the intervention program. In this sense, a meta-analysis identified that although there was a tendency to decrease IL-6, no significant reductions in IL-6 and TNF-α were observed in overweight/obese children or adolescents after a physical training program [32]. On the other hand, a variety of responses in terms of the levels of these variables have been reported; some studies reinforce these findings suggesting that, in the absence of weight loss, physical training does not improve the adipokines of obese youth [33,34,35], while a long-term physical training program (13 months) showed improvement in adipokine levels in overweight/obese adolescents [36]. Therefore, information regarding these variables is not established in the literature. In particular, IL-6 is a multifunctional cytokine that plays an important role in immune and inflammatory regulation and the anti-inflammatory effect is due to increased production of IL-6 and other micelles produced by the contracting muscle, whose kinetics are very fast, and increase up to one hour after exercise [37]. Therefore, in order to more clearly elucidate changes on this parameter after physical training, more recent studies have analyzed the acute effect of the first and last training session.

The analysis of the individual response showed that the number of responders regarding body fat, WC, and WHR were significantly higher in the IG than in the CG. Walsh et al. [38] reported similar results regarding body composition in which 72% were characterized as positive responders for body fat and 66% for WC of obese adolescents who participated in a combined physical training program. Likewise, the study by Yetgin et al. [39] showed improvement in CRF after a physical training program with obese male adolescents, although they did not report the prevalence of individual variability. It is known that the higher the CRF, the more protected the individual will be against the risk factors of obesity, reinforcing the importance of interventions that contribute to CRF improvements to exert a protective effect even in youth [40].

As far as is known, there is no evidence on the prevalence of responders in inflammatory markers after a multicomponent intervention consisting of exercise, nutritional, and psychological counseling with overweight/obese adolescents. Indeed, our data indicated unexpected results, in which the prevalence of responders was high for IL-6 and TNF-α in both CG and IG. The lack of previous studies that indicate the interindividual response to lifestyle intervention programs on inflammatory parameters makes difficult comparisons and assertions about the results found in this study, especially regarding the variables of IL-6, IL-10, and TNF-α. Furthermore, the presence of genetic polymorphisms may be associated with different responses found in the literature about these parameters [41].

Considering that there is wide inter-individual variability in the effects of interventions with physical training on health outcomes, it is important to note that genetic and environmental factors can contribute to this variability [42,43]. In addition, low sensitivity individuals, commonly considered unresponsive, require only a change in exercise mode, increased volumes and/or intensity, and improvements in measurement accuracy to generate a favorable response [42,44]. Thus, it is important to identify inter-individual variability and those who should exhibit a lower response to exercise to propose planned and effective interventions that contribute to the health and fitness of this population.

The literature is clear about the positive influence of CRF on body composition parameters and as a strong predictor for cardiovascular diseases and total mortality. In addition, an improvement in CRF is a potential mediator of the metabolic effects of exercise on adiposity and related comorbidities [45]. A higher level of CRF also appears to be more important for reducing the morbidity and likelihood of losing weight alone in the overweight and obese population [46]. 

Thus, seeking to understand the results obtained so far and the effects of this multi-component intervention aimed at improving physical fitness and body composition of overweight and obese adolescents, we verified the influence of the response of CRF on the other variables that showed a higher prevalence in the IG after the intervention. It was possible to identify that the changes in body composition variables (body fat, WC, and WHR) in the IG were associated with the CRF responders. Adolescents who were classified as responders to CRF showed a reduction in body fat (6.83%), WC (9.67 cm), and WHR (0.08 cm) compared to the non-responder’s peers. No studies were found comparing fitness changes between responders and non-responders after an intervention, which makes comparisons with our results difficult. In this regard, it is speculated that a frequency of three sessions of moderate to high intensity aerobic or combined physical exercise is sufficient to improve the CRF of overweight and obese adolescents [47]. In addition, a structured physical activity program appears to be more beneficial for improving CRF and reducing the percentage of fat in adolescents than just the practice of unstructured physical activity [7].

Multidisciplinary programs are considered the gold standard treatment for young people with obesity because interventions that propose behavioral changes, increased physical activity, and dietary education can be beneficial and effective in changing their lifestyle in a short time [48]. Besides, programs that provide combined exercise training can contribute to greater reductions in body composition parameters than isolated training [47]. Therefore, considering the aforementioned results, the multicomponent intervention program applied in the present study, including physical exercise, nutritional guidance, and psychological support was effective in increasing the physical fitness of adolescents and contributing to the improvement of body composition parameters. However, we did not find expressive results regarding inflammatory markers. 

In addition, these findings may have important implications in the design of interventions focused on the prevention/treatment of pediatric obesity and related comorbidities, such as low-grade inflammation, since the regular practice of moderate-intensity exercises direct the immune response to an anti-inflammatory state, such as the reduction of leptin levels promoted by this program, which is believed to be the main molecular mechanism desired to improve health outcomes [13].

Our study has some strengths. Indeed, we have highlighted the implementation of this multicomponent intervention program, which offered sports and physical activities, contributing not only to the physical health of adolescents but also in all dimensions of quality of life (physical, psychological, social, and environmental). In addition, as far as we are aware, this is one of the first studies that report the effects of the intervention in “mean terms” and the interindividual variability of inflammatory markers, as well as the influence of changes in CRF on body composition in a sample of overweight/obese adolescents. However, some limitations must be mentioned, such as the relatively small sample size due to the large number of dropouts during the intervention period, so that we can only detect the moderate effects and the quasi-experimental study design that requires caution regarding the extrapolation of these results. Besides, genetic factors or energy metabolism, which may be determinants of inter-individual variability, were not measured in the present study.

## 5. Conclusions

The results from this multicomponent intervention program, which went for 24 weeks and included physical exercise, nutritional guidance, and psychological support had a positive effect on body composition, physical fitness, and leptin reduction in overweight/obese adolescents. Furthermore, there was a higher prevalence of responders in the IG for body fat, WC, WHR, and CRF than in the CG, and reductions in some parameters of body composition were explained by CRF improvements. These findings reinforce the effectiveness of this program, however, studies with a larger sample size should be developed in order to establish that this intervention model can be adapted to school reality or social programs contributing to health promotion among overweight/obese adolescents.

## Figures and Tables

**Table 1:** Sample characteristics and comparison between control and intervention group at baseline.

	Control Group (*n* = 16)		Intervention Group (*n* = 17)		*p*	
Sex *	N (%)		N (%)			
Male	8 (50.0)		8 (47.1)		0.86	
Female	8 (50.0)		9 (52.9)			
Maturational stages *						
Not matured	5 (31.2)		1 (5.9)		0.10	
Continuing maturation	8 (50.0)		14 (82.4)			
Matured	3 (18.8)		2 (11.7)			
					**Anova**	
	**Mean (SD)**	**95% CI**	**Mean (SD)**	**95% CI**	**F**	***p***
Age (years)	13.13 (1.41)	12.37 to 13.88	13.00 (1.06)	12.45 to 13.55	0.08	0.77
Height (m)	1.56 (0.09)	1.50 to 1.60	1.60 (0.09)	1.55 to 1.65	2.03	0.16
Weight (kg)	66.40 (13.72)	59.09 to 73.71	72.63 (14.32)	65.26 to 79.99	1.62	0.21
BMI (kg/m^2^)	27.14 (3.37)	25.34 to 28.93	28.10 (4.38)	25.85 to 30.35	0.49	0.48
Body Fat (%)	28.16 (5.05)	25.47 to 30.85	31.50 (6.87)	27.96 to 35.03	2.49	0.12
WC (cm)	79.57 (7.53)	75.56 to 83.58	86.65 (11.28)	80.85 to 92.45	4.44	0.04
WHR (cm)	0.79 (0.06)	0.75 to 0.83	0.86 (0.07)	0.81 to 0.89	6.60	0.01
WHtR (cm)	51.13 (4.44)	48.76 to 53.49	54.06 (6.96)	50.48 to 57.64	2.04	0.16
Abdominal strength (rep)	22.63 (6.88)	18.96 to 26.29	20.65 (12.72)	14.11 to 27.29	0.30	0.58
Flexibility (cm)	21.15 (10.31)	15.66 to 26.65	20.44 (10.23)	15.18 to 25.70	0.04	0.84
CRF (m)	808.88 (153.27)	727.20 to 890.55	837.65 (168.60)	750.96 to 924.33	0.26	0.61
Leptin (ng/mL)	3.18 (1.81)	2.21 to 4.15	4.54 (2.33)	3.34 to 5.74	3.48	0.07
IL-6 (pg/mL)	15.04 (4.75)	12.51 to 17.58	18.71 (5.38)	15.94 to 21.48	4.27	0.04
IL-10 (pg/mL)	1.68 (1.56)	0.84 to 2.50	1.17 (0.37)	0.98 to 1.36	1.70	0.22
TNF-α (pg/mL)	57.03 (18.65)	47.09 to 66.96	61.65 (18.86)	51.95 to 71.35	0.50	0.48

* Data expressed as frequencies and percentages; chi-square test; data expressed as mean (standard deviation) and confidence intervals; One-way ANOVA test; significant differences for *p* < 0.05. CG: control group; IG: intervention group; BMI: body mass index; WC: waist circumference; WHR: waist to hip ratio; WHtR: waist-to-hip ratio; CRF: cardiorespiratory fitness; IL-6: interleukin-6; IL-10: interleukin-10; TNF-α: Tumor Necrosis Factor-Alpha.

**Table 2:** Intervention effect by group, time, and interaction on indicators of body composition, physical fitness, and inflammatory markers.

						GEE			Effect Size
		Pre	Post	Δ%	*p*	Group	Time	Interaction	Cohen’s d
BMI (kg/m^2^)	CG	27.13 (3.37)	26.40 (4.21)	−2.72	0.347	0.672	0.005	0.322	−0.52 *
	IG	28.10 (4.37)	26.55 (4.30)	−5.51					−0.48
Body Fat (%)	CG	28.16 (5.05)	28.30 (7.98)	0.49	0.002	0. 769	0.001	0.001	0.03
	IG	31.49 (6.87)	26.33 (8.63)	16.41					−1.03 **
WC (cm)	CG	79.57 (7.52)	79.36 (10.66)	−0.25	0.023	0.226	0.009	0.014	−0.02
	IG	86.65 (11.27)	79.31 (8.46)	−8.47					−0.81 **
WHR (cm)	CG	0.79 (0.06)	0.82 (0.08)	3.79	<0.001	0.652	0.058	<0.001	0.32
	IG	0.85 (0.07)	0.77 (0.04)	−9.30					−1.41 **
WHtR (cm)	CG	51.13 (4.44)	49.85 (5.76)	−2.50	0.035	0.625	<0.001	0.022	−0.24
	IG	54.06 (6.96)	48.50 (4.66)	−10.26					−0.93 **
Abdominal Strength (rep)	CG	22.63 (6.87)	22.00 (9.85)	−2.74	0.235	0.939	0.417	0.208	−0.09
	IG	20.65 (12.71)	23.53 (8.39)	13.94					0.30
Flexibility (cm)	CG	21.15 (10.31)	24.25 (9.21)	14.66	0.998	0.826	0.001	0.998	0.46
	IG	20.44 (10.23)	23.52 (10.62)	15.12					0.68 *
CRF (m)	CG	808.88 (153.27)	819.06 (175.04)	1.26	0.050	0.249	0.007	0.034	0.11
	IG	837.65 (168.59)	920.94 (202.27)	9.94					0.71 *
Leptin (ng/mL)	CG	3.18 (1.81)	3.06 (1.87)	−3.77	0.026	0.222	0.003	0.016	−0.09
	IG	4.54 (2.32)	3.29 (2.17)	−27.5					−0.85 **
IL-6 (pg/mL)	CG	15.05 (4.75)	12.03 (4.75)	−20.06	0.308	0.059	<0.001	0.279	−1.01 **
	IG	18.71 (5.38)	14.32 (5.22)	−23.46					−0.98 **
IL_10 (pg/mL)	CG	1.67 (1.55)	0.92 (0.62)	−44.91	0.138	0.393	0.040	0.125	−0.45
	IG	1.17 (0.37)	1.06 (0.38)	−9.40					−0.23
TNF-α (pg/mL)	CG	57.02 (18.64)	41.18 (15.84)	−27.78	0.071	0.058	0.014	0.052	−0.85 **
	IG	61.65 (18.85)	59.70 (28.90)	−3.01					−0.07

Data expressed as mean (standard deviation); data expressed as Δ%: percentage delta; Cohen’s *d*; * moderate *d* effect size (*d* > 0.5); ** large *d* effect size (*d* > 0.8); GEE: generalized estimating equations; *p*: significance level ≤0.05. GC: control group; GI: intervention group; BMI: body mass index; WC: waist circumference; WHR: waist to hip ratio; WHtR: waist-to-height ratio; CRF: cardiorespiratory fitness; IL-6: interleukin-6; IL-10: interleukin-10; TNF-α: Tumor Necrosis Factor-Alpha.

## Data Availability

The data presented in this study are available on reasonable request from the corresponding author.
